# An Innovative Multi-Model Neural Network Approach for Feature Selection in Emotion Recognition Using Deep Feature Clustering

**DOI:** 10.3390/s20133765

**Published:** 2020-07-05

**Authors:** Muhammad Adeel Asghar, Muhammad Jamil Khan, Muhammad Rizwan, Raja Majid Mehmood, Sun-Hee Kim

**Affiliations:** 1 Telecommunication Engineering Department, University of Engineering and Technology, Taxila 47050, Pakistan; adeel.asghar@students.uettaxila.edu.pk (M.A.A.); muhammad.jamil@uettaxila.edu.pk (M.J.K.); 2 Computer Science Department, University of Engineering and Technology, Taxila 47050, Pakistan; muhammad.rizwan@uettaxila.edu.pk; 3 Information and Communication Technology Department, School of Electrical and Computer Engineering, Xiamen University Malaysia, Sepang 43900, Malaysia; 4 Department of Brain and Cognitive Engineering, Korea University, Anam-dong, Seongbuk-ku, Seoul 02841, Korea

**Keywords:** brain–computer interface, convolutional deep neural network, deep feature clustering, EEG-based emotion recognition, feature selection, two-dimensional spectrogram

## Abstract

Emotional awareness perception is a largely growing field that allows for more natural interactions between people and machines. Electroencephalography (EEG) has emerged as a convenient way to measure and track a user’s emotional state. The non-linear characteristic of the EEG signal produces a high-dimensional feature vector resulting in high computational cost. In this paper, characteristics of multiple neural networks are combined using Deep Feature Clustering (DFC) to select high-quality attributes as opposed to traditional feature selection methods. The DFC method shortens the training time on the network by omitting unusable attributes. First, Empirical Mode Decomposition (EMD) is applied as a series of frequencies to decompose the raw EEG signal. The spatiotemporal component of the decomposed EEG signal is expressed as a two-dimensional spectrogram before the feature extraction process using Analytic Wavelet Transform (AWT). Four pre-trained Deep Neural Networks (DNN) are used to extract deep features. Dimensional reduction and feature selection are achieved utilising the differential entropy-based EEG channel selection and the DFC technique, which calculates a range of vocabularies using k-means clustering. The histogram characteristic is then determined from a series of visual vocabulary items. The classification performance of the SEED, DEAP and MAHNOB datasets combined with the capabilities of DFC show that the proposed method improves the performance of emotion recognition in short processing time and is more competitive than the latest emotion recognition methods.

## 1. Introduction

In recent years, much importance has been given to the recognition of human emotions using Electroencephalographic (EEG) signals. The Brain–Computer Interface (BCI), in Affective Computing, can be effectively used to control the devices with EEG signals [1]. With the development of machine learning tools, human emotions can be perceived more effectively using Neural Networks (NNs) compared to traditional handcrafted emotion recognition methods [2]. The task of identifying human emotions using EEG signals is still difficult due to the low temporal boundaries and the non-linear nature of the EEG signals, and different participants behave differently in the same scenario. In addition to neural networks, BCI provides a better approach to detect EEG signals for emotion classification [3]. EEG information is collected from the human skull using bio-amplifiers or electrodes. The more electrodes, the more useful information can be captured, while one electrode represents one EEG channel. Non-linear EEG signals for any subject and different channel numbers can have a dimensional curse to recognize a single emotion. To avoid the dimensionality issue and computational overhead, one should only select features that currently represent the user’s emotional state. To this end, much attention has been paid to recognizing emotions by converting one-dimensional EEG signals into two-dimensional spectral images. The spectral representation of the EEG signal contains information about time and frequency components. Feature selection is the process of selecting the most relevant features that efficiently predict system output and avoid dimensional curses. In emotion recognition and EEG-based classification, it is imperative to choose high-quality features because of computational overhead. In this article, we suggest a bidirectional feature selection method to save computational overhead using combined features of multiple neural networks. With the many advances in machine learning in recent years, high-quality features have been chosen using many traditional feature selection methods [4,5,6]. Since the EEG signal is non-linear, it is difficult to find high-quality feature properties due to its characteristics, accurately. The authors in [4] refer to a concept that the accuracy of classification may be reduced by adding more features to the learning algorithm after some time.

To reduce feature dimensions, a new approach is proposed to select channels and features that represent the highest emotional state. This article suggests a combination of differential entropy-based Channel Selection (CS) and Deep Feature Clustering (DFC) algorithms for feature selection using combined features of multiple Deep Neural Networks (DNNs) to reduce feature vector without interfering with the overall classification performance, in order to take full advantage of Bag of Deep Features (BoDF) [7] in reducing functionality on all channels of the EEG dataset—the first time this technique has been proposed to the best of our knowledge. Likewise, DFC used in this article to select features using selective channels from the EEG dataset. The significant difference between the two technologies is channel selection. Channel selection lowers computational costs and improves overall classification performance. Channel selection first reduces the number of channels by ignoring channels with low differential entropy and then uses DFC to obtain a new feature vector. Channel selection with DFC selects high-quality features from the four top-level neural networks. Three publicly available datasets are used to validate the proposed model. Differential entropy-based CS method selects only channels with high differential entropy while DFC methods select high-quality features and reduce the dimension of the feature vector. Support Vector Machine (SVM), k-Nearest Neighbour (k-NN) and Random Forest (RF) classifiers are used to classify emotional states.

### Contributions of the Work

So far, researchers recognized human emotions using a randomly selected number of channels. The computational speed can be increased, but the accuracy is reduced. The work’s contribution to the EEG-based literature on emotional perception can be summarized as follows:

- In this article, we have presented techniques for resizing and selecting high-quality combined features of multiple neural networks using Differential Entropy based Channel Selection and Deep Feature clustering (DFC). This ultimately reduces the feature vector size of the selective channel. The selection of channel provides excellent overall classification performance and helps to understand brain topology for emotional evaluation.
- Feature vectors are obtained from time-frequency representation of a series of EEG sentiment data pre-processed using Analytic Wavelet Transform (AWT). The proposed feature selection method for combined features of multiple neural networks, ordering features in a single matrix, and using the k-means algorithm to determine the vocabularies for each emotional state.
- Reduced feature vectors are classified for selected channels and features. For comparison, the accuracy of all channels and individual models is also verified on these datasets through classification performance. In the three datasets, the participants’ different emotional states are classified using all kernels of Support Vector Machine (SVM), k-Nearest Neighbors (k-NN) and Random Forest (RF) classifiers.

The rest of this article is structured as follows: Section 2 describes the literature on recognizing emotions, Section 3 describes the dataset and electrodes channel assignment. Section 4 introduces the emotion recognition framework and suggested depth of the functional model with SVM, k-NN and RF ratings. In Section 5, we describe the results of the experiments, also comparing different models for emotion perception. Section 6 concludes the article with future directions.

## 2. Literature Review

Emotion recognition is an essential part of brain-computer interference. Many researchers have attempted to categorize and detect everyday human emotions. It is proposed in [8] that features extracted can be classified using a support vector machine (SVM) with a Radial Basis Function (RBF) with 60% accuracy. Reference [9] extracted features from the EEG signal using the Dual-Tree Continuous Wavelet Transform (DT-CWT) based on time, frequency and nonlinear analysis. Four services from DT-CWT have trained the Simple Recurrent Unit (SRU) model. Correlation-based selection of subsets (CSS) helped to select the desired EEG signal characteristics. Higher-order statistics were used to classify the set of attributes in [10]. In reducing the dimensions, they concluded that CSS is more efficient than Principal Component Analysis (PCA) in terms of computational cost. The Empirical Mode Decomposition (EMD) method was used to parse the signal. The black hole algorithm that Meta-heuristics uses the EMD method to parse the signal to optimize the functionality of the Support Vector Machine [11]. According to [12], Ordinal Pattern Analysis (OPA) is more resistant to noise because it captures the repeating structure of a time series.

As machine learning tools progress, the demand for automatic recognition of human emotions increases [13]. The human emotional state is related to the perception and understanding of the participant. Disciplines such as human psychology, human cognition, computer science and artificial intelligence are heavily impacted by emotional awareness [14]. Emotion recognition becomes an essential part of providing people with emotion management as the demand for mobile applications increases. Wavelet-based feature extraction technology is suggested in [15] for classifying emotions in SEED dataset. They used flexible analytical wavelet transform (FAWT) for channel decomposition. They used SVM classifier on the SEED dataset to achieve 83.3% classification accuracy. Ref. [16] suggested a method for selection of evolutionary features, selecting the frontal laryngeal canal for classification and achieving 90% accuracy. Ref. [13], which uses MFCC (Mel Frequency Cepstral Coefficients) and reported an overall accuracy of 71.04% in the IEMOCAP dataset. In [17] multivariate empirical mode decomposition (MEMD) was used to reduce channel from 32 to 18. EEG signal is split into amplitude and frequency components called Intrinsic mode functions (IMFs). Two-dimensional sentiment states of arousal and valance are categorized by [17] using the SVM and ANN classifications. In other studies, parallax entropy is calculated from different EEG waves related to the EEG rhythm. The most effective rhythm for emotion recognition is beta and gamma waves [18]. Ref. [19] investigated the dynamic system functions of EEG measurements and other aspects important for cross-target emotion recognition (e.g., databases and sentiment analysis for different EEG channels). To eliminate repetitive features, the author’s suggested recursive method of Redundant Feature Elimination (RFE) reduces the size of features. A mean accuracy of 59.06% and 83.33% is achieved using DEAP and SEED dataset, respectively. In Ref. [20] author proposed the Level Feature Fusion (LFF) method to fuse 169 handcrafted feature together to form one feature vector. They claimed to provide good classification accuracy on MAHNOB dataset with SVM classifier with the fusion of different features.

Spectrum based measures were fused with OPA to achieve accuracy, and the results were improved up to 16%. Each node in a network works independently to represent features. As the system is divided into a hierarchy, the top layer collects these features and send it to mapping space to allow the system to perform further cognition [21,22]. In [23], balanced one-way ANOVA helped in Optimal EEG feature selection by calculating Hjorth parameters of different frequencies. The classification was done through a k-nearest neighbour, linear discriminant analysis, with Naive Bayes, SVM, and Deep Learning. Empirical Wavelet Transform (EWT) distributes the data in different empirical modes, and then autoregressive (AR) coefficients are calculated on desired nodes to form feature vector [17]. These feature vectors are provided to the classifier to recognize the emotions [24]. Accuracy of emotion detection lies in the generation of characteristic features. The authors of [25] also used EWT-based decomposition for the classification of EEG signals. Data adaptive EWT was proposed to improve the classification performance in motor imagery EEG signals. Least Square Support Vector Machine (LS-SVM) was used to achieve a classification accuracy of 95.2% and 94.6% in amplitude and frequency components, respectively.

Non-linear features of EEG, power spectral entropy, and dimension correlation were used by [26] for feature extraction. Emotions were induced with eight valence levels using the International Affective Picture System (IAPS) with similar arousal levels. Ref. [27] shows that Empirical Mode Decomposition (EMD) helps to decompose EEG signals to Intrinsic Mode Functions (IMFs). Classification accuracy on the DEAP dataset can be significantly improved by using domain adaptation techniques [28]. EMD extracted Intrinsic Mode Functions are analyzed by using Higher-Order Statistics (HOS) and Power Spectral Density (PSD). In Ref. [29] the classification of extracted features was done through the Naive Bayes Model, Linear Discriminant Analysis (LDA), and SVM. Long Short Term Memory (LSTN) recurring neural networks were used to train the machine [29]. Classification of EEG signals was done through Discrete Wavelet Transforms (DWT). This model was fused with the multichannel intelligent human emotion detection system. Mapping of emotions can be done using three-dimensional vectors: Valence, Arousal, and Dominance (VAD) [30].

Feature selection is a fundamental process to find a quality feature—the variety of features is essential to reduce dimensions with high classification performance. It helps to find the most prominent features in the classification space. In general, methods for scoring features can be divided into four classes: agreement-based, information theory, statistical-based, and sparse learning based [31]. So far, researchers have proposed several feature scoring methods, such as in [32,33]. In unsupervised feature selection, non-negative Laplacian is used to estimate the feature contribution [34]. In EEG-based emotion recognition, most authors use Principal Component Analysis (PCA) to reduce the feature dimension. PCA also selects functions based on their unique values. Omitting the features with an uncorrelated amount is a traditional cropping method. In reference, [35] authors suggest a dynamic search strategy to optimize a subset of statistical features. The feature is selected according to the Receiver Operating Characteristics (ROC) to determine the dominant features. They claimed to achieve better classification accuracy while reducing the size of the feature vector for electrocardiography (ECG) signal classification. Many studies claim to choose statistical characteristics. Likewise, in [36], the author used the Fisher Discriminant Ratio (FDR), so select features from the IMFs for chatter classification. Elements with high FDR values are selected for classification. In the traditional method of selecting a statistical feature, regardless of the metric used as an evaluation criterion, it is necessary to calculate the feature score for each dimensional feature and perform feature screening by sorting [6]. If the geometric dimension is very high, the calculation will take a long time. Ref. [37] proposes a method for selecting sparse emotion recognition modelling features. Ref. [25] used welch Power Spectral Density (PSD) to select 18 channels out of 118 to reduce processing time.

## 3. Materials

### 3.1. Electrode-Channel Positioning

The three datasets used in this work differ in the number of channels. EEG data from different participants were collected from all three datasets using standard electrode positioning known as the 10–20 International System [38]. The globally recognized system specifies the location of the electrodes and the cortical area, and “10′20” indicates that the gap between two adjacent electrodes is 10% or 20% of the total length of the head back and forth. Electrode-channel assignment helps to understand the behavior of the respective channel of different participants during recognition and feature selection process. The number of channel according to their respective electrode for SEED and DEAP dataset is represented in [7]. Electrode placement for DEAP and MAHNOB dataset is same due to same number of channels. Channel number refers to the number of electrodes used to receive one EEG signal from the skull. A total of 62, 32 and 32 channels are used to receive signals from the SEED, DEAP and MAHNOB datasets, respectively. The rest of the explanation is given in the next section.

### 3.2. Dataset I

Professor Bao Liang Lu governed experiments on human emotion using the BCMI Laboratory and provided a dataset of EEG signals, namely, SJTU Emotion EEG dataset (SEED) [39]. The gathering process of this dataset included 15 participants (eight females, seven males) who were subjected to watch 15 Chinese clips that would stimulate positive, negative, and neutral to a happy, sad and calm emotional states, using 10–20 Internationally standardized electrode placement scheme. The standard for selection from the clips is as follows:

- To avoid tiredness in the participants, the duration of the experiment should not be so long.
- The videos should be self-explanatory.
- A single target emotion should be evoked whilst watching the videos.

Each video had a time span of 4 min and was targeted to evoke a single emotion. The videos were designed to trigger coherent and meaningful emotions. The subjects underwent 15 tests for each experiment. A 5 s hint is given before the clip after the 4 min clip; there are 45 s for self-assessment and a 15 s rest in one sitting. A clip that might trigger the same emotions was not shown consecutively, and feedback was also taken on a questionnaire [39].

### 3.3. Dataset II

DEAP [40] dataset for emotion analysis using EEG, physiological, and video signals dataset is an on-line publicly available multi-model dataset for the human emotional behavior analysis. In total, 32 channel EEG and peripheral menstrual signals from 32 subjects were recorded while each item watched 40 highlighted videos of different category for 1 min. The signal was then downsampled to 128 Hz, and the noise was removed using bandpass and low pass filters. Four emotional states of Arousal, Dominance, Valence and Liking were assessed using self-assessment manikins (SAMs) [41]. SAM used to visualize the scale between 1 and 9. In this work, we mapped the scale in 4 levels of emotional behavior. The four emotional states are represented in two-dimensional Arousal and Valance scale, which are Low Arousal High Valence (LAHV) Alert, High Arousal Low Valence (HALV) Calm, High Arousal High Valence (HAHV) Happy, Low Arousal Low Valence (LALV) Sad [7].

### 3.4. Dataset III

MAHNOB [42] dataset collected by Professor Pantic and the iBUG group at Imperial College London and in part raised in collaboration with Prof. Pun and his team of University of Geneva, in the scope of MAHNOB project financially supported by the European Research Council under the European Community’s 7th Framework Program (FP7/20072013)/ERC Starting Grant agreement No. 203143. It is an also online available dataset for multi-model emotional behavior evaluation. The dataset is collected from 30 participants using bio-amplifiers placed on human skull [2] from 32 channels. During the acquisition of the signal, 12 video trials are presented to watch by the participants. Then they are asked about the video categories

**Figure 1:**
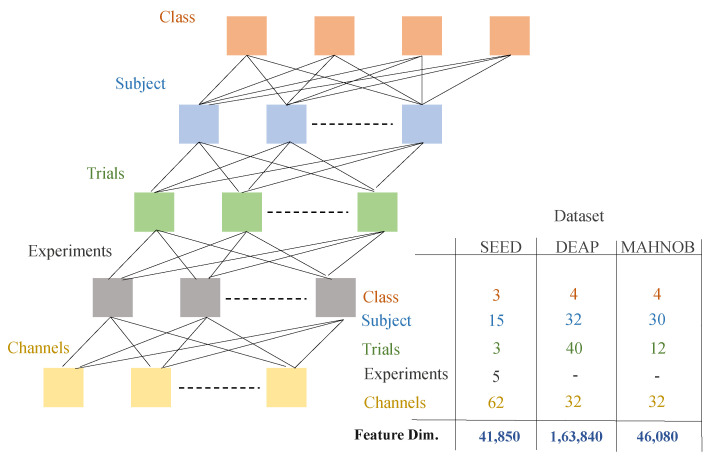
Feature dimensionality of datasets.

To select high-quality traits for EEG-based emotion recognition, three data sets were evaluated separately in this study using differential entropy-based channel selection and proposed DFC technique for feature selection. Figure 1 shows a feature vector tree of three datasets. The high dimension of the data set, up to the fully connected layer of the deep neural network, is visible from the figure.

## 4. Methodology

**Figure 2:**
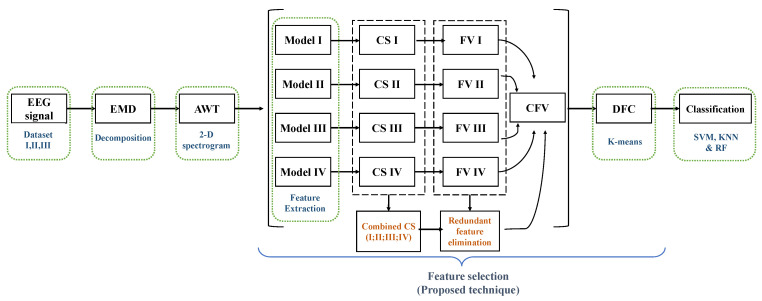
Framework.

Recognizing human emotions using EEG signals requires many procedures. High dimensions of the feature vector of the EEG signal is computationally expensive and has an extended processing time. Hence, it is necessary to reduce feature size and select high-quality features when training a network with good classification performance in a short time. As discussed earlier, the EEG signal is non-stationary, so the EEG signal is first decomposed using empirical mode decomposition to reduce the size of the feature. The parsed EEG signal is represented as a two-dimensional function of time-frequency. Spatial and temporal representation is achieved using flexible analytical wavelet transformations to attain all temporal boundaries. Features are extracted from four pre-trained neural networks and compare them to eliminate redundant features. In the proposed technique for selecting features for emotion recognition, the EEG signal is first decomposed and expressed as a spatial-temporal image. It then uses a combination of the functions of the four neural networks to select the right channel for recognizing emotional behavior. The chosen feature vector is obtained from the selected channel with Deep Feature Clustering. Figure 2 shows the general structure of this study. Frames are explained step by step in the upcoming sections.

### 4.1. Empirical Mode Decomposition

Empirical Mode Decomposition (EMD) based technology is used to parse the signal in various intrinsic mode functions called IMFs. The EMD methodology approaches rapid dissolution by calculating the maximum and minimum values of the signal. This reduces the quality of the output signal, but the features extracted from the method can yield significantly better results than traditional handcrafted emotion recognition methods. In the EMD methodology, after calculating the local maximum value M(x) and the minimum value m(x), the envelope v[n] is determined. The following steps demonstrate how EMD works. x[n] is the input signal and y[n] is the output of all IMF (n) plus x[n]=y[n].

1. Compute the maxima $M{\left(x\right)}_{i}$ and minima $m{\left(x\right)}_{j}$ values from the given EEG signal x[n].
2. Half the values of minima and maxima for fast processing:
  $\frac{M{\left(x\right)}_{i}}{2}$,$\frac{m{\left(x\right)}_{j}}{2}$
3. Compute eigen values of Maxima:
  $\xi_{i}\left(n\right)=f_{M}\left(M{\left(x\right)}_{i},n\right)$
  $x_{i}\left(n\right)=\sum_{i=1}^{N}\xi_{i}\left(n\right)$ where, *i* = 1,2 ... total number of samples *N*
4. Compute eigen values of Minima:
  $\xi_{j}\left(n\right)=f_{m}\left(m{\left(x\right)}_{j},n\right)$
  $x_{j}\left(n\right)=\sum_{j=1}^{N}\xi_{j}\left(n\right)$ where, *j* = 1,2 ... total number of samples *N*
5. Add both eigen values. $\xi_{k}=\xi_{i}\left(n\right)+\xi_{j}\left(n\right)$
  The resultant is the IMF of 1 EEG signal at *k* = 5.
6. Compute the signal envelopes e(n) alongwith mean $m\left(e\left(n\right)\right)=\frac{x_{i}\left(n\right)+x_{j}\left(n\right)}{2}$
7. If mean is equal to the IMF value than subtract it from the input y(n)=x(n)−m(e(n)) else m(e(n))=R(n).
8. Allowing shifting property to control the number of iterations. For this following formula is used.
  (1) $$y\left(n\right)=\frac{\sum_{t=0}^{N/2}{\left|m_{\left(t-1\right)}\left(n\right)-m_{t}\left(n\right)\right|}^{2}}{{m_{\left(t-1\right)}\left(n\right)}^{2}}$$
  *t* is the number of iterations where *t* = 1, 2, …, *N*/2, *k* is the samples from 1, 2, …, *N*−1 and *N* is the total number of samples.
  The algorithmic steps in EMD are similar to Bi and multi variate BEMD and MEMD EMD [17]. However, by reducing the number of extrema, it ultimately improves over all accuracy, reducing processing time.
9. IMFs is calculated The final equation becomes:
  (2) $$y\left[n\right]=\sum_{k=1}^{N-1}\xi_{k}\left(n\right)+R\left(n\right)$$
  where,
  ξ(n) is the IMFs of the original signal x(n) and R(n) is the residue.
  Envelopes are calculated using the *Sifting property* described in Equation (1).

### 4.2. Analytic Wavelet Transform

Analytical wave transformation (AWT) is used to obtain a complete representation of the time and frequency components. The literature argues that the most recent researchers have used DWT and STFT-based transformations. The reason for using AWT is that it has complete information about the time and frequency parts and can achieve a better representation compared to DWT. Besides, STFT-based transforms use custom windows, and AWT uses flexible windows depending on the signal. Besides, the AWT may be more suitable for non-linear signals compared to DWT and STFT due to the flexible wavelets. In AWT, a one-dimensional parsed EEG signal is displayed as a time-frequency component of a two-dimensional image known as a time-frequency representation (TFR). The TFR of the EEG signal is then scaled down for the extraction of features. The two-dimensional spectrogram of the input EEG signal represents the time and frequency components of the parsed EEG signal. The image for all datasets of selective channels are then fed to four different neural networks for feature extraction.

### 4.3. Feature Extraction

The TFR images are then fed to four pre-trained deep neural networks. Before extracting feature all the 2D spectrogram images are first resized to 224 × 224 × 3. The specifications of the four NNs are shown in Table 1.

#### 4.3.1. Model 1

As the name suggests, the abbreviation for ResNet is Residual Network, and a new network of learning resources is being introduced. The deep convolutional neural network has changed the elements for image classification. Resnet-50 has multiple stacked layers and is ready to use network [43]. This is the main theory of the residual learning model. Training for this type of system is more comfortable than setting up a shallow and straightforward rotating neural framework, and it also eliminates the problem of infected accuracy. The depth of the residual network is 50 layers. The ResNet-50 model consists of 5 levels with convolution, maximum pooling and ReLu layers. ResNet-50 has over 23 million learnable parameters. It uses skip connection to add the output from the previous layer to the next layer, which helps to reduce the problem of the gradient disappearing. In a fully connected FC layer, 1000 features of each channel are captured for each participant and trial. So in this network we get the feature vector of 41,850 × 1000, 163,840 × 1000 and 46,080 × 1000 for SEED, DEAP and MAHNOB dataset, respectively.

#### 4.3.2. Model 2

The feature extraction process was also performed by a convolutional DNN called GoogLeNet. GoogLeNet is a multilayer DNN consisting of 22 layers [44]. GoogLeNet uses 5 million parameters compared to the previous ResNet-50 with 23 million parameters. Thus, it can also be provided in the main limitations of memory, and computation costs [45]. The two-dimensional spectrogram image was delivered in 22 layers and used a convolution and maxi pooling process. The convolutional layer in the Inception Model uses a rectified linear activation function. In the RGB colour space, the average value of the network is 224 × 224. The collapsed layer consists of 1 × 1 filters called “3 × 3 reduce” and “5 × 5 reduce” used before the convolutions of 3 × 3 and 5 × 5.

The rectifying function is elaborated as

(3) $$ReLU=max\left(0,x\right)$$

The selection of maximum value is made between 0 and input x. We used up till the fully connected layer of “loss3-classifier” which provided the output of 1000 attributes. Figure 1 provides information about the output feature dimension vector of the three datasets. As stated earlier, the SEED dataset employs three classes by performing five trails on 15 subjects, DEAP and MAHNOB datasets employ four classes, each. The number of channels that were used is 62 for each subject (Refer to Table 2). The EEG signals are perceived as images after the 2-D representation. These 62 images are made for each of the 62 individuals using TFR with 1000 dimensions of the loss3-classifier layer. So the obtained feature vector from the all three datasets are 41,850 × 1000, 163,840 × 1000 and 46,080 × 1000.

#### 4.3.3. Model 3

Inception V4 [46] is googLeNet’s advanced multi-layered network. Inception v4 is a combination of three residual blocks and one ensemble block to reduce computational cost. As with other networks, Inception V4 consists of two parts. Fully connected and classified layers. Therefore, we only performed feature extraction to omit the classification hierarchy.

#### 4.3.4. Model 4

The fourth model we used is VGG-16 [47]. The three fully connected (FC) layers follow a convolutional layer (with different depths in different architectures) are stacked. The configuration of the fully connected layer is the same in the above-discussed networks. All hidden layers are equipped with rectification (ReLU) non-linearity. Since one (except one) of the networks does not contain local response normalization (LRN), such normalization does not improve the performance of the dataset but instead increases memory usage and computation time.

We have not used the classification layers of all four models, only for in-depth extraction of features. The choice of all models is based on their layers and parameters. The first three models were chosen because they skip the network, accelerating the time for non-linear signals. The fourth model was chosen because it has 1000 attributes that are comparable to the first three selected models.

### 4.4. Differential Entropy-Based Channel Selection

After extracting features from all four models, the next step is to select channels where differential entropy is used. All channels in each dataset are not aware of the user’s full emotional behavior. This step also helps to choose high-quality features for human emotion recognition. Differential entropy-based channel selection selects high entropy channels and omits others.

The channel selection process is essential in omitting irrelevant and redundant features while maintaining the quality of the selected features. By using effective methods to extract quality attributes, the total number of attributes is significantly reduced without compromising classification standards. A general decision-making algorithm is used in this paper to evaluate the relevance of a subset of characteristics. This allows us to successfully display ambiguous data in the boundary portion of that characteristic subset. The recommended algorithm uses differential entropy to evaluate a subset of functions to get high-quality channels [48].

The idea of the differential entropy is the compartment and all functions caused by a specific features subset [49,50]. By implementing this uncertainty measure, plenty of useful channels can be attained significantly.

The variation of the information between a feature subset and the full feature set is expressed through the entropy measure. In this way, it gives an approach to measure the discernibility over the information embedded in the original data.

Note that for any P⊆C and xϵU, there is ${\left[x\right]}_{C}\subseteq {\left[x\right]}_{P}$

(4) $$E\left(P|U⊕C\right)=-\frac{1}{U}\sum_{x\epsilon U}log_{2}\frac{\left|{\left[x\right]}_{C}\right|}{\left|{\left[x\right]}_{P}\right|}$$

Therefore, for P⊂B⊂C, the following properties of the differentiation entropy hold:

(5) $$E\left(B|U⊕C\right)\leq E\left(P|U⊕C\right)$$

(6) $$E\left(B|U⊕C\right)=E\left(P|U⊕C\right),\frac{U}{P}=\frac{U}{B}$$

The threshold value of 1.145 is defined, and channels that exceeded that threshold were selected. Using the differential entropy-based channel selection methods (26, 8 and 12) are selected from 62, 32 and 32 of all three datasets, respectively. Channel selection greatly helps in reducing the combined feature vector (CFV). CFV is a unified feature in all four models. The feature vector obtained after channel selection are 17,550 × 1000, 40,960 × 1000 and 17,280 × 1000 in each dataset I, II and III. The selected number of channels for SEED dataset are 1,3,4,5,7,8,11,12,14,15,18,21,23,26,27,29,32,35,25,38,48,52,60,43,58 and 42, for DEAP dataset 9,10,12,13,20,21,23 and 24 and for MAHNOB dataset 5,7,19,25,30,31,32,20,14,18,22 and 18 are selected channels. Refer to [7] for their corresponding channel positions.

#### Combined Feature Vector (CFV)

To select a high-quality feature, feature vector obtained from all four DNN models are first concatenated. First of all, redundant features are removed using the euclidean distance. The scope of the feature vector has been significantly reduced to save training time on data size. Previously [51], only 8 or 12 channels were used by researchers, and the remaining channels were ignored, resulting in poor accuracy. In this article, we proposed a differential entropy-based channel selection that selects high-quality features for fast processing with excellent overall classification performance. The Combined feature vector, redundant feature elimination of all datasets is given in Table 2.

Feature vector (FV) is obtained by extracting features from each model. Since FV has 1000 attributes in each model, we have linked features which are CFV=FV×4. Redundant feature elimination (RFE) reduces the size of features by eliminating unnecessary elements. Therefore, the feature dimension after RFE is 30,521 × 1000 for SEED, 52,850 × 1000 for DEAP, and 32,914 × 1000 for MAHNOB dataset. Table 2 shows the dimension of feature vector at every stage.

### 4.5. Deep Feature Clustering

**Figure 3:**
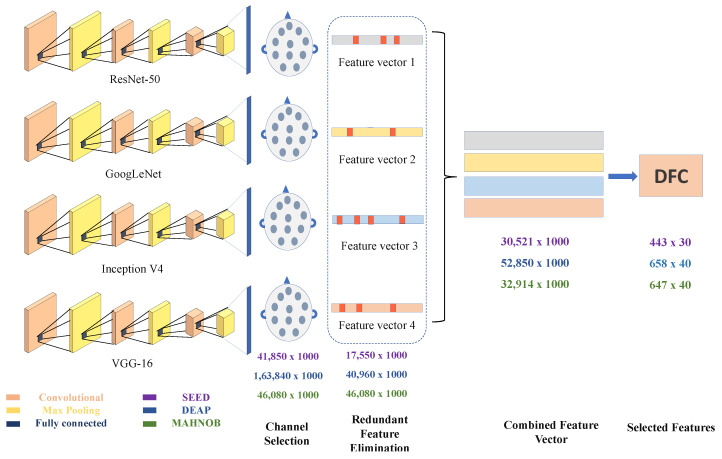
Detailed proposed method (feature selection process).

The motivation to propose Deep Feature Clustering (DFC) is the use of a bag of words (BoW) in machine vision [52]. Vocabulary assignment in BoW is a low ranking of image classification features. Bag of Deep Features (BoDF) [7] used to cluster features from all channels. The clustering of features is used to reduce feature dimension. Likewise, in this case, we use DFC, as suggested in this paper, to select high-quality features. Unlike BoDF, DFC method clusters contain the selected EEG dataset channels. The architecture of DFC is somehow similar to BoDF technique but major difference is in channel number. The detailed architecture of the proposed feature selection method is shown in Figure 3. Selected channel feature vectors and CFVs are supplied to DFC for selection of high-quality features and reduction of the size of the feature vector. The RFE feature vector is supplied to the DFC for further feature reduction. The proposed DFC model consists of two steps. Step 1 involves grouping data using k-means clustering. Similar features are considered as one feature after clustering. Step 2 calculates the histogram using the deep cluster function.

#### 4.5.1. Clustering

In step 1 of DFC, RFE data of CFV are grouped using k-means clustering. The k-means algorithm is suitable for large data sets, but there are problems with other available clustering techniques. Over-fitting occurs when processing large data sets [53]. K means that the algorithm has clustered the features for each class as a function of k. For datasets I, II and III of class 3, 4 and 4, respectively. The total number of cluster functions is k×class. Because there is no universal truth for choosing k values, it is selected using hit and trial method. First, k is chosen as an arbitrary value, and the distance from the object to the centre is calculated. This process is repeated for different values of k until all features are clustered correctly. This process of grouping similar features is known as vocabulary assignment. The vocabulary of the given CFV is calculated at different values of k>2. Then we used to calculate the sum of squared error between correct and targeted values. The sum of the squared error is calculated using difference equation to find features which are wrongly clustered. Starting from k=2 the difference equation is used to calculate the sum of the squared errors. Wrong clustering of features will result in large error rate. To select the *k* value, different experiments was performed. At different values of k>2 we found that at k=10 the sum of the square error is minimal. So, according to k×class for each data set, the vocabulary sizes are 30, 40 and 40 For each of the datasets I, II and III.

#### 4.5.2. Histogram of Features

Histogram vocabulary characteristics are calculated from the original EEG dataset, the histogram calculation will help in gathering subject independent features. It refer to the number of single-channel features that appear in the original feature vector of each class. The visual vocabulary compares each feature vector to the EEG data set of all selected channels. For the SEED dataset, the functions are used sequentially, compared to 26 channel features, and the frequency of occurrence is counted. For each attribute, we get a histogram feature of 443 × 30. Accordingly, the histogram feature of the DEAP data set is 658 × 40 and the MAHNOB data set is 647 × 40. The scope of function is significantly reduced using the DFC technology.

### 4.6. Classification

The selected and combined feature vector were classified using the SVM [54] k-NN [55] and RF [56] classifiers. The high-quality feature vectors of 443 × 30, 658 × 40 and 647 × 40 are obtained using the proposed model. Each attribute vector is classified with three classification techniques, and the classification accuracy is outstanding. The results are also compared with other studies using the same datasets.

In addition, the classification accuracy of each network was also calculated; the combined results were also obtained and displayed. The result table shows a variant of classification accuracy for Model 1, Model 2, Model 3, Model 4 and combined model using cubic kernel (best efficiency) in the SVM and k-NN classifier for SEED, DEAP and MAHNOB dataset. The performance evaluation of all three classifiers is discussed in the next section.

## 5. Results and Discussion

The performance of the proposed EEG-based emotion recognition process is validated on both the datasets for benchmarking. Selected features are then classified with Support Vector Machine (SVM) and k-Nearest Neighbor (k-NN) to measure the performance of the proposed scheme.

### 5.1. Support Vector Machine Classifier

The SVM classifier is used for optimal classification. As discussed earlier, we did not use the classification layer of the DNN model. The DNN used in this paper can perform better ranking for up to 1000 classes, while SVM can efficiently classify for a small number of classes. In our case, the total number of classes is 3 for SEED, 4 for DEAP and 4 for MAHNOB. Using all SVM kernels, it appears that the cubic kernel outperforms the best classification performance. The principle of the SVM classification is based on aggregation [54]. Hyperspace is optimized for separating data classes with enclosed space. During the training, the decision boundaries of each class are calculated, so the number of classes is separated. Another reason to use SVM that it is suitable for nonlinear classification is [57]. When all datasets are classified as SVM, this provides the best classification accuracy of 97.5% in the SEED data set while utilizing the capabilities of DFC.

**Figure 4:**
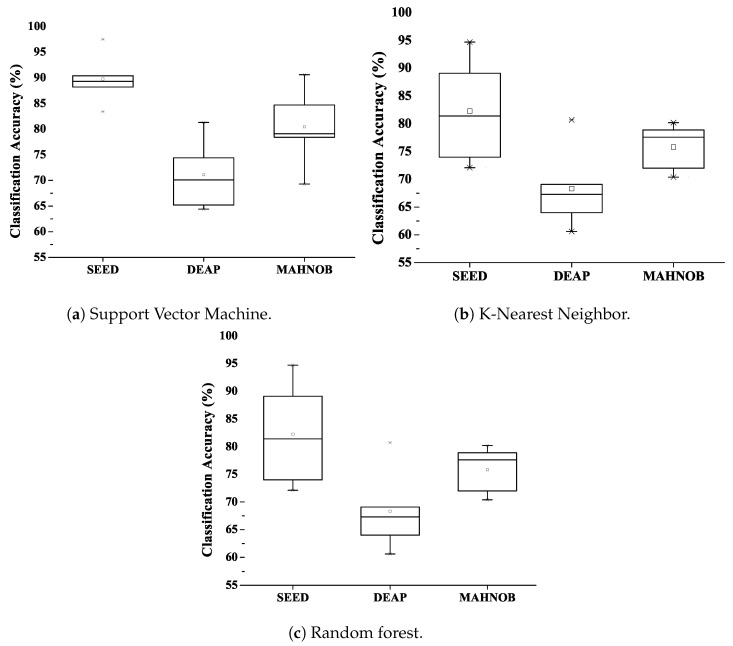
Classification performance of three datasets.

The classification result of SVM classifier of all three datasets with DFC and without DFC is shown in Figure 4a.

### 5.2. k-Nearest Neighbour Classifier

Second, classify the proposed model using the k-NN classifier. The k-NN classification classifies objects based on the values of the nearest neighbour function. It calculates the distance of each attribute value from each class. This algorithm uses the Euclidean distance method to find the nearest neighbour. Classification performance was achieved with a fine kernel. Best classification accuracy of 92.3% was achieved in the SEED dataset and 78.9% and 77.3%, respectively, in the DEAP and MAHNOB datasets. Figure 4b shows the performance statistics of k-NN using the combined DFC and individual models.

### 5.3. Random Forest Classifier

The random forest (RF) classification is a discriminatory classification. The use of RF is motivated by its computational efficiency. It consists of several trees that function as ensembles. This will considerably reduce the chance of errors. When the proposed DFC technology is classified using RF, the classification accuracy achieved is 94.7% in a SEED dataset, slightly larger than the k-NN classifier. We achieved an accuracy of 80.7% in the DEAP dataset and 80.2% in MAHNOB, which is shown in Figure 4c using deep feature clustering.

### 5.4. Training a Network

The multi-model method of extraction and selection of features is performed on the GTX 1080 GPU with 8 GB of RAM. It turns out that training multiple networks at the same time, with nearly 1.2 million layers is too big compared to a single GPU. So it extracts features from different models at different times, removes duplicate features and then combines the extracted features. Feature selection is crucial because it reduces computational costs. This is done using the proposed DFC technique. After obtaining high-quality features. The feature vector can now be learned in seconds on a simple core i3 or higher processor. On GPUs with or without DFC, the run time of each cycle is shown in the Table 3. You can see that selecting features with DFC significantly, shortens processing time and improves overall rating performance.

### 5.5. Evaluation Parameters on Proposed Feature Selection Method

#### 5.5.1. Cost–Entropy Function

The cost function [58] evaluated the performance measurement of the proposed technique. The cost function gives the error rate between the target value (t) and the actual value (a). Following equation is used to calculate the cost entropy function.

(7) $$Cost=-\frac{1}{t_{p}}\sum_{c=1}^{C_{n}}\left(\left[t_{c}*ln\left(a_{c}\right)+\left(1-t_{c}\right)*ln\left(1-a_{c}\right)\right]\right)$$

where,

$t_{p}$ is the training traits
*t* is the targeted value
$C_{n}$ is the number of classes
*c* is the class label/index
*a* is the actual value

The proposed DFC network training model was adopted in Matlab 2019b on the Windows operating system. Training a multi-model DFC method requires significant calculation and processing time. The attribute vectors obtained for 10× fold validation used during training setup. Indicates that splitting data do not overlap. To reduce errors in the cost function, we used the gradient descent algorithm as an optimization function with a learning rate of 0.001. The Adam optimizer was used in the experiments to training the proposed model, and the fewer number of iterations were used to reach the optimal point. The results obtained using DFC and without DFC are presented in Table 4 and Table 5.

#### 5.5.2. *t*-Test and F-1 Score

We used *t*-test parameter to test whether the selected feature is relatively useful. The *t*-test calculates the average difference between the DFC and the chosen element from the original feature vector. The higher the value, the more significant the difference. Mathematically, the *t*-test establishes the problem description by assuming a null hypothesis that the two means are equal by taking samples from each of the two sets. Based on the applicable formula, absolute values are calculated and compared with the default values, and the null hypothesis is accepted or rejected accordingly. Greater *p* represents the feature vector obtained are more likely to occur in original feature vector.

The F1 score is used to measure the accuracy of the test and balances precise measure and sensitivity. The F1 score can use both precision and sensitivity to deliver test performance with more realistic measurements. The f-1 score is the function of precision and sensitivity or recall. Hence it provides the information if the data is clustered or selected unevenly. The F-1 score is calculated as:

(8) $$f-1score=2*(\frac{P*S}{P+S})$$

where, *P* is the Precision and *S* is the sensitivity. Both of them are calculated using the following equations.

(9) $$P=\frac{1}{N}\sum_{c=1}^{N}\frac{TP(c)}{TP(c)+FP(c)}$$

(10) $$S=\frac{1}{N}\sum_{c=1}^{N}\frac{TP(c)}{TP(c)+FN(c)}$$

*N* is the total number of class, *c* is the class index, *TP* is true positive, *FP* false positive and *FN* is the false negative.

#### 5.5.3. Mutual Information and Pearson Correlation

Mutual information gathers the information or correlation present between adjacent feature values under same emotional state. It selects the highly correlated features. Let *X* and *Y* be two feature values, then MI(X;Y) is calculated using equation.

Mutual information is measured by calculating the combined probability density function (PDF).

p(X) and p(Y) is the probability of two variables then its PDF will be

(11) $$MI\left(X;Y\right)=\sum_{x\in c}^{C}\sum_{y\in c}^{C}p\left(x,y\right)log\left(\frac{p(x,y)}{p(x)p(y)}\right)$$

*X* and *Y* are two vectors from different classes with the same feature. *c* is the class index and *C* is the total number of classes. The smaller the *MI* value, the more significant the difference between the two attribute values. Therefore, attribute values are removed from the final attribute vector. Only attribute values with high *MI* values are selected. This process tests high-quality features with all emotion information. If the attribute vector decreases, the computational costs also decrease.

This analysis enabled us to find changes in the functional values after and before DFC. MI is the mutual information between classes of feature vectors. The smaller MI, the higher the inter-class difference of feature vector and vice versa. In the Pearson correlation, distances between inter-classes and intra-classes are calculated for DFC attribute values. The minimum distance between inter-class represents the values of the same class, and the maximum difference within the intra-class represents the values of other classes. This parameter was evaluated for the proposed technique, and the selected characteristic values turned out to be the best for emotion recognition using DFC.

**Figure 5:**
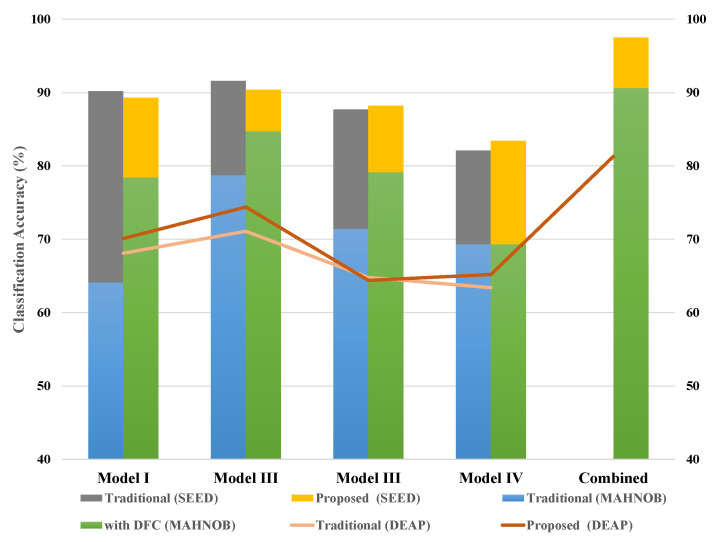
Comparison of proposed model with traditional model in classification accuracy using SVM classifier.

The following Figure 5 shows the difference between using DFC and the existing DNN model for classification. The graph shows that the combined feature vectors have achieved good classification accuracy and that the use of DFC can significantly reduce computational cost without compromising overall classification performance. Therefore, selecting a high-quality feature will shorten the processing time and achieve excellent classification performance.

The proposed model, concerning the comparison table, has a higher classification accuracy than previous studies. The classification accuracy obtained from the SVM classifier is higher compared to emotion recognition using k-NN and RF classifiers (see Table 6). The proposed DFC benchmarking technique has been compared to recent emotion recognition techniques that deliver results on SEED and DEAP datasets.

Feature reduction based on the proposed Feature selection technique shows that using clustering of multiple neural networks to obtain one feature vector increases classification accuracy. Choosing high-quality features helps train neural networks in a short time with excellent classification performance. According to the results of all three datasets, the DFC model selects superior quality features for EEG-based emotion recognition.

## 6. Conclusions

DNN’s success has already been proven to be competent in various classification tasks, especially in the context of image classification. So to classify EEG data, we combine the features of four pre-trained DNN architecture. We have taken the necessary steps to reconstruct the input data into a two-dimensional image to feed and train network. This work proposed an efficient and innovative method of high-quality feature selection to recognize human emotions effectively. High-quality features based on DFC are selected from a large feature table to shorten the training time of the network. In this article, the fists of all raw EEG signals are decomposed to reduce noise in non-linear EEG signals, and we performed wavelet-based transforms to convert the one-dimensional decomposition EEG signals into two-dimensional time-frequency representations. The feature values taken from the four models are combined with having a single feature vector and introduced to reduce the size of the feature vector. In the proposed scheme, the number of channels is reduced using differential entropy. Subsequently, deep feature clustering was introduced to achieve high-quality features. The proposed DFC model is designed to use publicly available datasets SEED, DEAP and MAHBOB. Reduced attribute vectors, when validated with SVM, k-NN and RF classifiers, provide excellent classification performance. The results demonstrate that the method of selecting features improves the overall classification performance while lowering computational cost. Selected characteristics of high quality have shown to exhibit emotional states compared to available datasets. The selected DFC features show less computational cost than the conventional CNN features and the traditional hand-crafted features. The average classification accuracy of 97.5% is achieved for three states of the SEED dataset, 81.3% for four states of the DEAP dataset and 90.6% for four states of the MAHNOB dataset.

The proposed model has dramatically improved the features for extracting features. Classification performance has improved significantly over previous models. Feature selection using DFC also provides a gateway for real-time emotion recognition through EEG. This work suggests how to combine data using multiple neural networks and design an associated deep learning model. Future research undertakings will explore the effects in the emotional analysis framework through the combination of various neural networks.

## Figures and Tables

**Table 1:** Conventional Deep Neural Network (DNN) architecture.

	Name	Layers	Parameters	FC Attributes
Model 1	ResNet-50	50	23 million	1000
Model 2	GoogLeNet	22	6.7 million	1000
Model 3	Inception V4	48	5 million	1000
Model 4	VGG-16	12	138 million	1000

**Table 2:** Combined feature vector.

Stages	Dataset I	Dataset II	Dataset III
FV	41,850 × 1000	163,840 × 1000	46,080 × 1000
after channel selection FV	17,550 × 1000	40,960 × 1000	17,280 × 1000
CFV	70,200 × 1000	163,840 × 1000	691,20 × 1000
after RFE CFV	30,521 × 1000	52,850 × 1000	32,914 × 1000

**Table 3:** Execution time on datasets with combined DNN.

Dataset	Model	Traditional Execution Time (s)	Proposed Method Execution Time (s)
**I**	ResNet-50	8360	432
	GoogLeNet	2247	144
	Inception V4	6920	252
	VGG-16	10,160	612
	Combined	-	216
**II**	ResNet-50	15,000	756
	GoogLeNet	3565	252
	Inception V4	9680	576
	VGG-16	16,791	972
	Combined	-	324
**III**	ResNet-50	9381	648
	GoogLeNet	2612	180
	Inception V4	8000	396
	VGG-16	3042	720
	Combined	-	216

**Table 4:** Performance evaluation without DFC.

Traditional Methods														
			ResNet-50			GoogLeNet			Inception v4			VGG-16		
			Acc(%)	F1	*t*-Test	Acc(%	F1	*t*-Test	Acc(%)	F1	*t*-Test	Acc(%)	F1	*t*-Test
**Classifier**	**SVM**	**D I**	81.1	0.77	p=0.130	84.2	0.81	p=0.130	83.2	0.81	p=0.130	77.7	0.72	p=0.130
		**D II**	68.1	0.65	p=0.350	71.1	0.66	p=0.350	64.8	0.61	p=0.350	63.4	0.62	p=0.350
		**D III**	64.1	0.62	p=0.001	78.7	0.73	p=0.001	71.4	0.70	p=0.001	69.3	0.60	p=0.001
	**k-NN**	**D I**	81.9	0.79	p=0.430	73.4	0.71	p=0.430	71.7	0.69	p=0.430	77.9	0.77	p=0.430
		**D II**	63.1	0.61	p=0.210	74.1	0.72	p=0.210	75.8	0.75	p=0.210	61.8	0.56	p=0.210
		**D III**	81.5	0.79	p=0.110	84.0	0.80	p=0.110	71.2	0.71	p=0.110	73.5	0.70	p=0.110
	**RF**	**D I**	69.9	0.58	p=0.810	72.1	0.71	p=0.810	69.5	0.51	p=0.810	71.3	0.71	p=0.810
		**D II**	62.1	0.61	p=1.000	65.7	0.61	p=1.000	68.5	0.62	p=1.000	61.1	0.60	p=1.000
		**D III**	71.9	0.61	p=0.090	76.1	0.70	p=0.090	70.3	0.55	p=0.090	62.9	0.58	p=0.090

**Table 5:** Performance evaluation with DFC.

Using DFC																	
			ResNet-50			GoogLeNet			Inception v4			VGG-16			Combined		
			Acc(%)	F1	*t*-Test	Acc(%)	F1	*t*-Test	Acc(%)	F1	*t*-Test	Acc(%)	F1	*t*-Test	Acc(%)	F1	*t*-Test
**Classifier**	**SVM**	**D I**	89.3	0.77	p=1.00	90.4	0.89	p=1.00	88.2	0.81	p=1.00	83.4	0.82	p=1.00	97.5	0.96	p=1.00
		**D II**	70.1	0.70	p=0.89	74.4	0.71	p=0.89	64.4	0.62	p=0.89	65.2	0.60	p=0.89	81.3	0.81	p=0.89
		**D III**	78.4	0.71	p=0.91	84.7	0.80	p=0.91	79.1	0.78	p=0.91	69.3	0.60	p=0.91	90.6	0.91	p=0.91
	**k-NN**	**D I**	88.1	0.85	p=1.00	85.7	0.84	p=1.00	78.3	0.77	p=1.00	79.0	0.77	p=1.00	92.3	0.90	p=1.00
		**D II**	74.4	0.74	p=0.87	77.0	0.75	p=0.87	62.3	0.61	p=0.87	69.3	0.67	p=0.87	78.9	0.78	p=0.87
		**D III**	71.1	0.74	p=1.00	72.6	0.69	p=1.00	75.4	0.72	p=1.00	77.1	0.75	p=1.00	77.3	0.78	p=1.00
	**RF**	**D I**	81.4	0.70	p=0.91	89.1	0.71	p=0.91	74.0	0.71	p=0.91	72.1	0.69	p=0.91	94.7	0.84	p=1.00
		**D II**	64.0	0.61	p=0.91	69.1	0.65	p=0.91	60.0	0.59	p=0.91	67.3	0.67	p=0.91	80.7	0.77	p=0.91
		**D III**	77.6	0.71	p=1.00	78.9	0.75	p=1.00	72.0	0.69	p=1.00	70.4	0.61	p=1.00	80.2	0.85	p=1.00

**Table 6:** Comparison with recent emotion recognition techniques.

Reference	Feature Type	Dataset Used	Channel Used	Classification	Accuracy (%)
[8]	MEMD	DEAP	10	SVM	60.00
[9]	DT-CWT	SEED	62	SRU	80.02
[9]	DT-CWT	SEED	62	SVM	71.43
[9]	DT-CWT	SEED	62	k-NN	68.52
[10]	Higher order statistics (HOS)	own	34	SVM	82.20
[13]	MOCAP	IMOCAP	62	CNN	71.04
[14]	MFM	DEAP	18	CapsNet	68.20
[15]	MFCC	SEED	12	SVM	83.50
[15]	MFCC	SEED	12	RF	72.07
[15]	MFCC	DEAP	06	RF	71.10
[17]	MEMD	DEAP	12	ANN	75.00
[17]	MEMD	DEAP	12	k-NN	67.00
[18]	STRNN	SEED	62	CNN	89.50
[19]	RFE	SEED	18	SVM	90.40
[20]	LFF	MAHNOB	all	SVM	68.75
[26]	International Affectve Picture System (IAPS)	DEAP	06	RF	71.10
[7]	BoDF	SEED	62	SVM	93.80
[7]	BoDF	SEED	62	k-NN	91.40
[7]	BoDF	DEAP	32	SVM	77.40
[7]	BoDF	DEAP	32	k-NN	73.60
Proposed method	Combined DNN	SEED	26	SVM	97.50
Proposed method	Combined DNN	SEED	26	k-NN	92.30
Proposed method	Combined DNN	SEED	26	RF	94.70
Proposed method	Combined DNN	DEAP	08	SVM	81.30
Proposed method	Combined DNN	DEAP	08	k-NN	78.90
Proposed method	Combined DNN	DEAP	08	RF	80.70
Proposed method	Combined DNN	MAHNOB	12	SVM	90.60
Proposed method	Combined DNN	MAHNOB	12	k-NN	77.30
Proposed method	Combined DNN	MAHNOB	12	RF	80.20

## Link to the Code

https://github.com/Ad-collab/Emotion-Classification.git.

## Abbreviations

The following abbreviations are used in this manuscript:

BCI	Brain–Computer Interface
EEG	Electroencephalogram
DNN	Deep Neural Network
DFC	Deep Feature Clustering
CS	Channel Selection
CFV	Combine Feature Vector
EMD	Empirical Mode Decomposition
AWT	Analytic Wavelet Transform
TFR	Time-frequency representation
SVM	Support Vector Machine
k-NN	k Nearest Neighbor
RF	Random Forest
SAM	Self-Assessment Manikins
